# Gambling and other addictive behaviors among higher education students in Finland—insights from a large-scale survey

**DOI:** 10.3389/fpsyg.2025.1529051

**Published:** 2025-03-26

**Authors:** Jussi Palomäki, Sari Castrén, Niko Männikkö, Tiina Latvala

**Affiliations:** ^1^Finnish Institute for Health and Welfare, Helsinki, Finland; ^2^Department of Digital Humanities/Cognitive Science, University of Helsinki, Helsinki, Finland; ^3^Social Sciences Department of Psychology and Speech-Language Pathology Turku, University of Turku, Turku, Finland; ^4^Department of Medicine, University of Helsinki, Helsinki, Finland; ^5^Research Unit of Health Science and Technology, Faculty of Medicine, University of Oulu, Oulu, Finland; ^6^Centre for Research and Innovation, Oulu University of Applied Sciences, Oulu, Finland

**Keywords:** gambling, behavioral addictions, students, socioeconomics, compulsive internet use

## Abstract

**Introduction:**

Problem gambling forms a continuum of severity from mild to moderate and severe. While most young individuals who gamble do so responsibly, for some gambling becomes a problem with severe negative consequences. Excessive gambling is strongly linked with substance use and other behavioral addictions, particularly among students.

**Methods:**

In this study we draw from a large sample of higher education students to evaluate the links between gambling behavior, socioeconomic status, mental health, alcohol use and other potentially addictive behaviors. We analyzed our data using post-stratification survey weighted logistic regression modeling.

**Results:**

Our main findings were that (i) students in polytechnics were more prone to gamble and experience harms than university students, (ii) self-perceived problem gambling was significantly associated with alcohol use but not with drug use or smoking, and (iii) compulsive internet use predicted gambling problems but not increased gambling activity.

**Discussion:**

Our results underscore the need for early detection of harmful behaviors among students, and early interventions for those with severe problems. Student health checks should be used to screen for harmful gambling habits and difficulties in handling finances.

## Introduction

For many young individuals, gambling is a popular pastime both online and offline (Emond et al., 2022; Hollén et al., 2020; Riley et al., 2021). For some, however, excessive gambling becomes a problem and leads to financial debt and significantly reduced mental and physical well-being (Armitage, 2021; Montiel et al., 2021; Oksanen et al., 2018). Problem gambling forms a continuum of severity, ranging from mild to moderate and severe (Delfabbro, 2013), and is strongly linked with substance use, other behavioral addictions as well as neurocognitive problems (Ford and Håkansson, 2020; Goudriaan et al., 2006; Hayatbakhsh et al., 2012). Generally, problem gambling is a behavioral pattern characterized by excessive gambling with significant negative consequences and loss of control. Worldwide problem gambling prevalence rates among adolescents and young adults range between 0.2 and 12.3% (Calado et al., 2017). In Finland, about 5% of 18–24- and 25–34-year-olds were gambling at a problem level in 2019 (Salonen et al., 2020). In the current fifth edition of the Diagnostic and Statistical Manual of Mental Disorders (the DSM-5; American Psychiatric Association, 2013), Gambling Disorder (GD) is used to denote severe forms of problem gambling diagnosed by a physician, and the same term (GD) is used also in the revised version of World Health Organization’s International Classification of Diseases (11th revision; ICD-11; World Health Association, 2018).

It is well-known that young age, male gender, exposure to gambling advertising and experienced loneliness increase and exacerbate problem gambling behavior (Freund et al., 2022; Riley et al., 2021; Sirola et al., 2019, 2023). Due to various factors such as impulsivity, students are highly susceptible to risky behaviors, gambling included, which may result in several adverse consequences both in terms of psychological and financial well-being (Shen, 2023; Zolkwer et al., 2022).

Moreover, problem gambling is strongly associated with family background and socioeconomic status (Hahmann et al., 2021; Halladay et al., 2020). Lower socioeconomic status (e.g., lower education and income) is associated with a higher likelihood of gambling problems, and students with lower school success have reported more severe psychological consequences of gambling (Livazović and Bojčić, 2019). In addition, individuals with low income, particularly those living in poverty, are at high risk of developing problems with gambling. These individuals, compared with individuals with high income, are more likely to take loans placing them in financial stress, which, in turn, can lead to developing a gambling disorder (Castrén et al., 2018; Hahmann et al., 2021; Håkansson, 2020; Latvala et al., 2019).

The relationship between socio-economic status and gambling behavior is complex and varies depending on contextual factors. Research suggests that individuals with lower SES may be at higher risk for problem gambling due to financial strain, higher neighborhood gambling availability, and the perception of gambling as a way to improve financial situations (Langham et al., 2016). Additionally, gambling may serve as a coping mechanism for economic hardship, reinforcing a cycle of financial instability (Koomson et al., 2022).

On the other hand, studies have also linked higher SES to increased gambling participation, particularly in contexts where gambling is seen as a form of recreation or social activity (MacDonald et al., 2004). Individuals with greater financial resources may engage in gambling more frequently but experience different levels of risk for developing gambling-related harm. These mixed findings suggest that SES alone may not fully explain problem gambling, necessitating a theoretical framework to better understand its role. Here we apply Jessor’s Problem Behavior Theory (PBT) (Jessor, 1991) to conceptualize problem behaviors, such as gambling, as arising from the interaction between individual characteristics, environmental influences, and behavioral tendencies. According to PBT, gambling can be understood as part of a broader pattern of risk-taking behavior that is influenced by social and economic contexts. PBT is particularly relevant for young adults, as this life stage is marked by increased autonomy, changing financial responsibilities, higher education, and evolving social influences—all of which can shape gambling behavior.

Excessive- and problem gambling are positively correlated also with other harmful behaviors that share similar features. These include, but are not necessarily limited to, excessive use of social media, gaming, online shopping and pornography use, and, more generally, compulsive internet use (Burleigh et al., 2019; Charzyńska et al., 2021; Müller et al., 2022; Tullett-Prado et al., 2023). Some researchers suggest these harmful behaviors should collectively be defined as behavioral addictions (Brand et al., 2019). Others, however, highlight the need for more high-quality and wide-ranging data (e.g., epidemiological, neurobiological, psychological, and clinical) to determine how behavioral addictions are defined (Griffiths, 2022). While the true nature of behavioral addictions is being debated, such behaviors are nonetheless clearly linked with negative life consequences and reduced well-being, especially among young adults.

In addition to the strong intercorrelation between various behavioral addictions, there is a well-known bi-directional link between problem gambling and substance use: Individuals with one of these behaviors are at a higher risk of developing the other. Several studies have identified that substance use disorders are typical comorbidities for problem gambling behavior (Buja et al., 2019; Caldeira et al., 2017; Castren et al., 2021; Ford and Håkansson, 2020). Evidence thus supports the co-occurrence of addiction for both substances and various behaviors [i.e., having a behavioral addiction increases the likelihood of developing another addiction (Marmet et al., 2019)]. Moreover, those who experience co-occurring problematic and addictive behaviors are at higher risk of poor mental health (e.g., depression) and physical health (Håkansson and Karlsson, 2020; Jolly et al., 2021; Karlsson and Håkansson, 2018). Indeed, this may lead to a cycle of reciprocity, wherein mutual exacerbation occurs between two or more problematic behaviors (Angioletti and Balconi, 2022; Carbonneau et al., 2023), especially among young people.

One way to gain knowledge on why young individuals succumb to problem gambling, other behavioral addictions, or harmful substance use, is to draw data from different student institutions. Among young individuals, there are large differences in risky behaviors between students in vocational institutions and students in high schools. Students in Finnish vocational institutions, compared with high school students, sleep and exercise less, have unhealthier eating habits, and are more likely to smoke cigarettes, binge drink, and abuse drugs. Similar differences are apparent also between students in the polytechnics and students in the universities, polytechnics students being more at risk for various risky behaviors (hereafter we refer to polytechnics and universities as “study sectors”^1^; Kataja et al., 2022; Ollila and Ruokolainen, 2022). The parents of university students have, on average, higher socioeconomic status (including level of education and income) than the parents of polytechnics students (Nori et al., 2021). The student profiles in the polytechnics tend to range from poorly achieving students of highly educated parents, to highly achieving students of parents with lower education, more so than in the universities (Heiskala et al., 2021). Thus, the “two study sector system model” (universities vs. polytechnics) appears to separate well-achieving students into either universities or polytechnics depending on the students’ family backgrounds, with universities being the typical choice for students with well-educated parents.

While there are clear differences in substance use between students in the two study sectors (universities vs. polytechnics), research is scarce on such differences in gambling behaviors, or other potentially harmful and addictive behaviors. This is a clear gap in our current knowledge, given the well-established link between gambling and substance use on the one hand, and gambling and other behavioral addictions on the other. Our primary objective is to disentangle the effects of educational socioeconomic factors (e.g., study sector) from the effects of comorbid substance use and other potentially behavioral addictions. We focus on three levels of self-reported gambling activity: whether the respondents have gambled at all, whether they have gambled actively on a weekly basis, and whether they thought gambling posed a problem for them. Our analysis is driven by the research question of whether these three levels of gambling engagement are best explained by socioeconomic factors, substance use, other potentially addictive behaviors, or self-reported general health. The results can inform the development of effective preventive efforts for those who may be at a greater risk of harm.

## Materials and methods

### Participants and procedure

The study is based on the health and well-being research carried out in 2021 by the National Institute for Health and Welfare in Finland. The data collection was carried out in February–March 2021 during the third wave of the coronavirus pandemic. Informed consent was obtained from all participants, and the study was approved by the Ethics committee of the Finnish Institute for Health and Welfare. All methods were performed in accordance with the relevant guidelines and regulations. The study invitations were sent to 11,912 undergraduate students aged 18 to 34 by email who were randomly selected from all Finnish higher education institutions, which had an overall population of 100,216 (Universities) and 96,977 (Polytechnics) students. The proportion of respondents in the total data was 52.5%, yielding a final sample size of 6,258 (38.2% males). The response rates varied by gender and age, being 60.1% for females and 43.7% for males.

### Measures

#### Dependent variables (DVs)

Our dependent variables were dichotomous (yes/no) questions on (1) whether participants had gambled at all, (2) whether they perceived gambling at a problematic level, and (3) whether they had gambled on a weekly basis during the past 12 months. The DVs 2 (self-perceived problem gambling) and 3 (weekly gambling) were evaluated on categorical scales but dichotomized in the analyses due to skewed response distributions. Thus, the DVs were not based on existing validated scales, but rather three separate one-item questions. Of the respondents, during the past 12 months, 2,212 (37.9%) had gambled at least once, 361 (6.2%) had gambled on a weekly basis, and 224 (3.8%) perceived having a gambling problem.

#### Independent variables

We assessed problematic internet use using a short version of the Compulsive Internet Use Scale (CIUS-5) comprising 5 items rated from 0 “never” to 4 “very often” (Lopez-Fernandez et al., 2019). The scores of the scale range from 0 to 20, with higher scores corresponding to a higher severity of problematic internet use; however, we use the item-wise mean score in our analyses. The original version of CIUS has demonstrated adequate factorial, content, and concurrent validity, and good reliability (Meerkerk et al., 2009). Cronbach’s alpha for the CIUS-5 scale in this study was 0.783. Employing a general measure such as the CIUS-5 to assess problematic internet use is vital, as it provides a comprehensive overview of internet use behaviors and their potential impact on an individual’s life (Fineberg et al., 2025). This broad measure helps to identify patterns of problematic use and enables an initial screening and understanding the extent of internet-related challenges. However, it is equally important to incorporate additional questions focused on specific online activities, such as video gaming and social media. These targeted questions yield deeper insights into areas where problematic behaviors may be particularly pronounced.

Alcohol consumption was evaluated using the dichotomized AUDIT-C scale (Bush et al., 1998). AUDIT-C has 3 items (evaluated on a 5-point Likert scale) and is used to identify hazardous drinkers or those with active alcohol use disorders (including alcohol abuse or dependence). The scores for each item were summed participant-wise, and cut-off points recommended by Kaarne et al. (2010) were used to define risky drinking among males (score ≥ 6) and females (score ≥ 5). Participants’ use of cigarettes and snuff (moist cut tobacco that can be loose or pouched and placed in the mouth) was evaluated on a 5-item Likert scale: (1) Not at all, (2) Previously but have since stopped, (3) Less than once a week, (4) Weekly but not daily, (5) Daily. The responses were dichotomized into 1 = “Has used” and 0 = “Has not used.” Use of other drugs was measured by a single question: “Have you used X at least once in the last 12 months?” where X is replaced by a list of substances (cannabis, ecstasy, amphetamine/methamphetamine, cocaine, drugs and alcohol together, drugs in order to intoxicate), and likewise dichotomized into 1 = “Has used any substance in the past 12 months,” 0 = “Has not used any substance in the past 12 months.”

Mental well-being was measured using the 12-item General Health Questionnaire (GHQ-12; Goldberg, 1978). Each item assesses the severity of recently experienced mental health problems using 4-point Likert-scales (from 0 to 3). Scores were averaged item-wise and reverse coded so that higher scores indicate better health. Cronbach’s alpha for the scale was 0.901.

Participants reported on their sociodemographic- and background characteristics including gender (male/female), age [recoded categorically as (i) 18–22, (ii) 23–26, (iii) 27–30, and (iv) 31–34, but analyzed as a continuous variable], income [categorical: “How did you manage financially during the past 12 months? (1) Very well, (2) Well, (3) I managed but had to live sparingly, (4) My income is low and uncertain”; reverse-coded so that higher scores reflect better income status], self-perceived loneliness (5-point Likert, analyzed as a continuous variable: “Do you feel lonely? (1) Never, (2) Very rarely, (3) Sometimes, (4) Somewhat often, (5) All the time”), and study sector (university/polytechnic). Participants were also asked, using dichotomous yes/no questions, whether they felt they had a problem with using social media, gaming, internet porn, or online shopping. Finally, participants were asked if they had ever taken quick loans or consumer credit (0 = “have not taken,” 1 = “have taken but have not have problems paying back,” 2 = “have taken and have had problems paying back”).

### Statistical analysis

All analyses were conducted using the R platform for statistical computing (v. 4.2.1, R Core Team, 2013). Survey weights were calculated based on national statistics on age, gender, study sector as well as credits obtained during the past semester. For more details on data collection and the survey sample weights, see Parikka et al. (2022).

We used survey-weighted (post-stratification weighting) multiple logistic regression with the survey package in R (Lumley, 2004). The post-stratification weights were calculated using the inverse probability weighting (IPW) method. Registry data available for the entire population on age, gender, mother tongue, study sector, and study credits for the previous semester were used as predictors for missing participation (see Parikka et al., 2022 for full details on the sampling and weighting process). In the logistic regression models the independent variables were gender, age, income, risky alcohol consumption (AUDIT-C), problematic use of (i) social media, (ii) gaming, (iii) internet porn, (iv) online shopping, perceived loneliness, general mental health (GHQ-12), problematic internet use (CIUS-5), previous use of snuff, cigarettes, drugs, and previous use of quick loans or consumer credit.

The fitted multiple logistic regression model satisfied the assumptions of linearity of logit-values for continuous variables. The generalized variance inflation factor (GVIF) values ranged between 1.04 and 2.23, suggesting there were no issues of multicollinearity.

Given our relatively large sample size, missing values across all variables (21.3% in total) were omitted listwise. Therefore, in our final analyses the sample size ranged between 5,149 and 5,151 participants (depending on the dependent variable). As a robustness check we also analyzed our data by imputing all missing values using predictive mean matching (PMM) with the mice R package (Van Buuren and Groothuis-Oudshoorn, 2011). PMM is widely used, and it typically imputes more plausible values than other imputation methods, since it draws real values sampled from the data as replacements for the missing values. Five separate datasets were imputed, models were fit separately for these imputed datasets and the results were thereafter pooled. The results largely mirrored the results from the analyses using listwise deletion, confirming their robustness; thus, we report only the analyses with missing values omitted listwise.

For effect size estimates we use Cragg-Uhler and McFadden pseudo-r^2^ -values. In addition, we calculated the average binary cross-entropy loss based on a 5-fold cross-validation method for complex sample surveys (Wieczorek et al., 2022).

## Results

Gambling participation (having gambled at least once in the past 12 months) was predicted by male gender, older age, lower income, studying at a polytechnic (as opposed to university), risky alcohol use, having used (moist) snuff, having taken quick loans, and loneliness. Overall, the model had pseudo r^2^-values of 0.16 (Cragg-Uhler) and 0.09 (McFadden) and a 5-fold average binary cross-entropy loss of 0.63 (SE = 0.005). We note again that age was measured categorically but analyzed as a continuous variable, with the highest age category being 31–34 years.

Weekly gambling (having gambled at least weekly during the past 12 months) was predicted by male gender, older age, studying at a polytechnic, risky alcohol use, having taken quick loans, and reporting problematic gaming (based on a single dichotomous item). The pseudo r^2^-values were 0.14 (Cragg-Uhler) and 0.11 (McFadden) and a 5-fold average binary cross-entropy loss of 0.254 (SE = 0.009).

**Figure 1:**
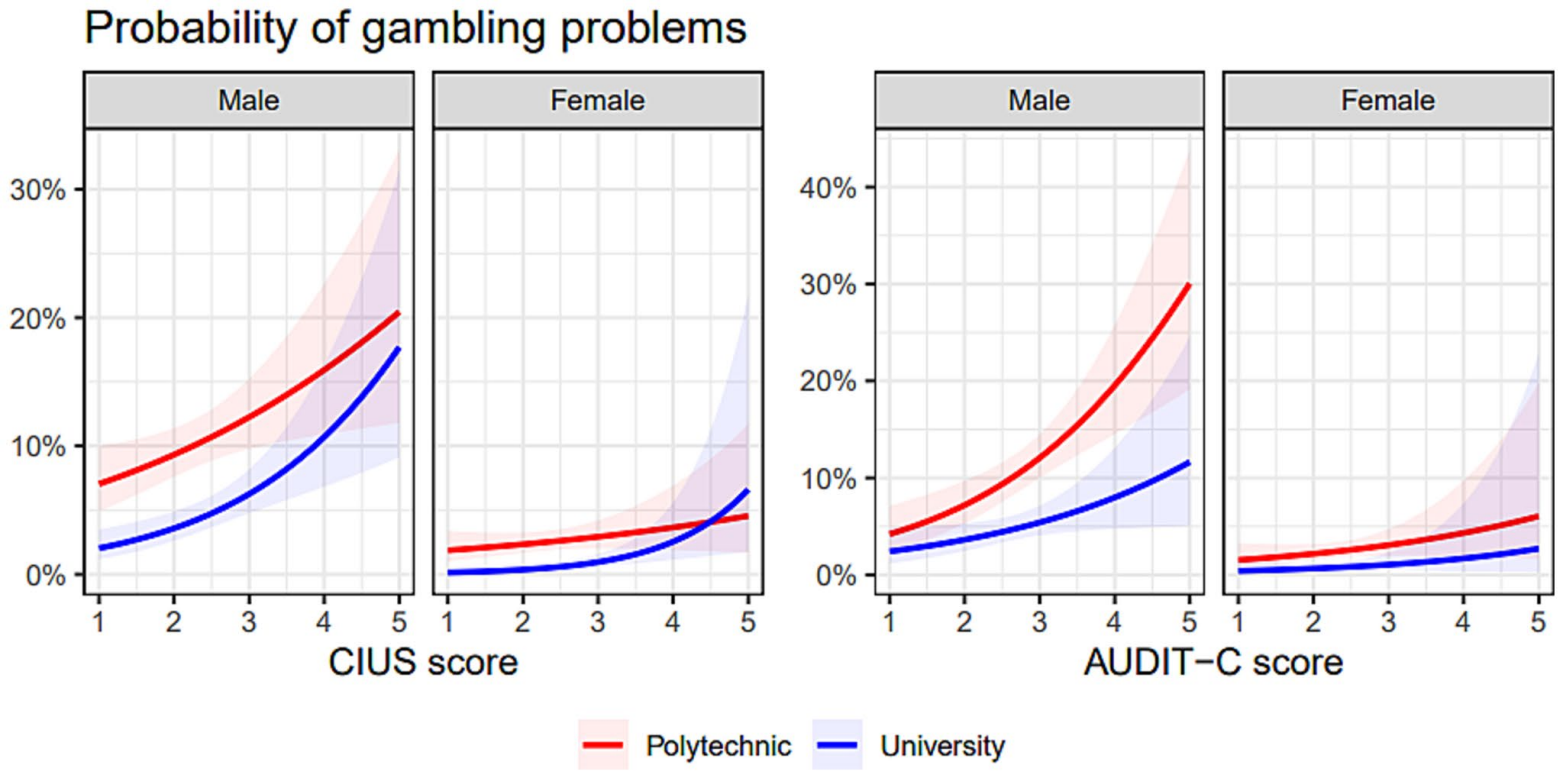
The association between probability of self-perceived gambling problems and CIUS scores (left) and AUDIT-C scores (right), separately for male and female respondents as well as Polytechnic- and University students. In the Results section, AUDIT-C scores were dichotomized, but here visualized as a continuous variable for clarity. The slopes are predictions from a weighted logistic regression model (without control variables) with 95% confidence bands colored.

Finally, reporting gambling being a problem during the past 12 months was predicted by male gender, better income situation, studying at a polytechnic, risky alcohol use, higher CIUS scores, having used snuff, and having taken quick loans. Reporting problematic internet pornography use was negatively associated with self-perceived problem gambling. The pseudo r^2^-values when predicting self-perceived problem gambling were 0.21 (Cragg-Uhler) and 0.18 (McFadden) and a 5-fold average binary cross-entropy loss of 0.155 (SE = 0.008). See Table 1 for full details of the analyses, and Figure 1 for visualizations.

**Table 1:** Multiple logistic regression model with past 12-month (i) gambling participation, (ii) weekly gambling, and (iii) self-perceived problem gambling (yes/no) as the dependent variables in separate models.

Dependent variable	Odds ratio/*t*-value		
	Gambling participation	Weekly gambling	Self-perceived problem gambling
Independent variable			
*(Intercept)*	1.18/0.51	0.08/−4.05***	0.07/−3.22***
Gender (ref: Male)	0.47/−10.58***	0.28/−8.93***	0.16/−8.58***
Age	1.11/2.85***	1.41/5.05***	0.99/−0.1
Income	0.92/−2.11*	1.01/0.25	0.77/−2.68**
Study sector (ref: University)	1.64/7.52***	1.67/4.05***	2.08/4.24***
AUDIT-C (ref: Not at risk)	1.14/5.22***	1.34/2.19*	1.88/3.73***
GHQ	1.15/1.85	1.07/0.50	0.94/−0.29
CIUS	0.95/−0.96	0.99/−0.08	1.42/2.85**
Smoking (ref: No)	1.28/1.76	0.98/−0.08	1.01/0.04
Drug use (ref: No)	1.14/0.19	0.88/−0.71	0.82/−0.92
Snuff use (ref: No)	1.87/4.32***	1.27/1.15	1.72/2.43*
Quick loans (ref: Have not taken)			
*Have taken, no trouble paying back*	2.41/4.86***	2.33/3.43***	3.38/4.21***
*Have taken, trouble paying back*	2.23/3.41***	3.24/3.81***	4.71/4.54***
Loneliness	0.88/−3.21***	0.88/−1.58	1.01/0.11
Social media (ref: No)	0.97/−0.33	1.03/0.19	0.76/−1.1
Online gaming (ref: No)	0.96/−0.27	1.56/2.24*	1.44/1.52
Online pornography (ref: No)	0.99/−0.06	0.64/−1.54	0.42/−2.34*
Online shopping (ref: No)	1.13/0.76	1.15/0.41	2.01/1.78
Model fit			
Cragg-Uhler pseudo r^2^	0.15	0.14	0.21
McFadden pseudo r^2^	0.09	0.11	0.18
5-fold average binary cross-entropy loss (SE)	0.630 (0.005)	0.254 (0.009)	0.155 (0.008)

*p < 0.05, **p < 0.01, ***p < 0.001. Statistically significant cells for predictors highlighted in shades of grey (except the intercept), darker shades meaning more significant.

## Discussion

In this study, drawing from a large and representative sample of higher education students, we explored the associations between gambling behavior, socioeconomic status (including study sector), mental health, alcohol use, and other potentially addictive behaviors. We found that both gambling participation and weekly gambling – that is, having gambled at least once, or weekly, during the past 12 months – were associated with male gender, studying at a polytechnic (as opposed to a university), risky alcohol use, having taken quick loans, and older age. We note, however, that age had a narrow range in our study (from 18 to 34 years in four categories). For young individuals and students in particular, gambling behavior is influenced by several contextual factors such as academic pressure, social dynamics and possible financial hardships. While typically young age is linked with excessive gambling, such findings are based on analyses with a much wider range of age. Our findings highlight that for students gambling behavior may involve complex social dynamics between individuals with different life circumstances, which should be noted when catering potential interventions to different age groups (Bramley and Fitzpatrick, 2022).

Snuff use, lower income, and not having experienced loneliness were also significantly associated with gambling participation, but not with weekly gambling, and we observed a weak positive association between experiencing problems with gaming and weekly gambling (see also André et al., 2022). Finally, significant predictors of students’ self-perceived gambling problem during the past 12 months were male gender, lower income, studying at a polytechnic, risky alcohol consumption, compulsive internet use, having used snuff, having taken quick loans, and not having used online pornography (albeit the association with online pornography was weak).

Our findings are largely consistent with previous studies demonstrating links between problem gambling, lower socioeconomic status, and comorbid problematic or compulsive use of alcohol, other substances, and the internet (Baggio et al., 2017; King et al., 2020; Marchica et al., 2017; Nsereko et al., 2023; Raybould et al., 2021; Samuelsson et al., 2018; Wardle, 2019), but there are some key differences. We found that students at polytechnics, compared with university students, were significantly more likely to participate in gambling, perceive having gambling problems, consume alcohol at a risky level, take on quick loans, and use the internet compulsively. Prior research has consistently identified substance use (e.g., drugs, alcohol, and tobacco) as a risk factor for problem gambling across age groups (Hayatbakhsh et al., 2012). In our current study, however, we found that smoking and using drugs (other than snuff) were not strongly implicated in self-reported gambling participation, weekly gambling, or self-perceived gambling problems overall when all other variables were controlled for, which is not in line with previous work (Emond et al., 2022; Jiménez-Murcia et al., 2021). We did find a strong link between having taken quick loans – regardless of whether the student had trouble paying it back – and all three gambling variables. This finding resonates with earlier work showing that hoping to win money often motivates individuals, including students, to gamble (Hagfors et al., 2023), and that financial losses together with social isolation are linked with problem gambling behavior (Koomson et al., 2022). While having taken quick loans was identified as a predictor, it could also be a consequence of problem gambling behavior. Taking quick loans may indicate that a person is chasing after losses or wins, which exacerbates gambling and results in increased financial losses over time (Nigro et al., 2022; Lister et al., 2016). The data in the current study were collected during the height of the Covid-pandemic, which may have exacerbated feelings of loneliness among the students (Sirola et al., 2023).

One possible explanation for our finding on the effects of self-reported loneliness is that certain forms of gambling, particularly social or recreational gambling, provide opportunities for social interaction. For example, casino visits, poker games, and sports betting are often social activities, allowing individuals to connect with peers and engage in shared experiences (e.g., Laakasuo et al., 2014; Palomäki et al., 2021). Thus, individuals with lower levels of loneliness may be more likely to gamble due to their active participation in social gambling environments.

Additionally, our finding aligns with research indicating that gambling motivations vary depending on individual and contextual factors. Some individuals gamble primarily for excitement and entertainment, rather than as a response to negative emotional states (Macey et al., 2024). This perspective is consistent with Jessor’s Problem Behavior Theory (PBT), which suggests that risk behaviors, including gambling, are influenced by broader lifestyle patterns, social contexts, and peer networks. In this sense, gambling may be embedded within a broader social and recreational lifestyle, particularly among those who experience lower levels of loneliness. Future research could further explore the social dimensions of gambling participation, distinguishing between problematic gambling behaviors and socially motivated gambling. Finally, examining gambling subtypes (e.g., solitary vs. social gambling) could provide deeper insights into how loneliness interacts with different gambling motivations.

Interestingly, compulsive internet use was not significantly associated with gambling participation or weekly gambling but was a highly significant predictor of self-perceived gambling problems. It is well-known that increased gambling activity is a major risk factor of problem gambling (Allami et al., 2021; Harris et al., 2021). As an exploratory analysis, we retained self-perceived problem gambling as the dependent variable and added weekly gambling as a predictor (alongside all other predictors). This did not dilute the association between compulsive internet use and self-perceived problem gambling (in fact it strengthened it), suggesting that increased gambling activity is not the reason why compulsive internet use predicts problem gambling. Thus, using the internet in a compulsive manner is associated with potentially harmful gambling regardless of the amount of time spent gambling (Rouvinen et al., 2023).

Problematic internet use may also inadvertently serve as a platform for online gambling, given the rapid rise of information and communication technologies that have transformed the gambling landscape. With the growing accessibility of online gambling sites, active internet users are increasingly drawn to this digital environment, as highlighted by Gainsbury et al. (2015). Popular forms of online gambling, such as poker, online casino games, and bingo, have found a receptive audience among those with elevated levels of internet engagement (Biolcati et al., 2015; Griffiths et al., 2010). This suggests that individuals who exhibit problematic internet behaviors may be more susceptible to the allure of online gambling, as these platforms offer not only entertainment but also the potential for social interaction and immediate gratification. Consequently, the convergence of excessive internet use and online gambling raises important questions about the impact of digital media on gambling behaviors and the need for appropriate interventions to address this growing concern. Future research should attempt to clarify and disentangle the links between compulsive internet use, gambling activity, and problem gambling (Karlsson et al., 2019); notably, the conceptual overlap between measures of problem gambling and problematic internet use should be investigated (Dahl and Bergmark, 2020).

Our study has a few noteworthy limitations. The reliance on self-assessment via single-question measures for certain constructs in this study is inherently limited. While self-assessment tools can effectively capture subjective experiences, they are prone to biases such as social desirability, recall bias, and individual differences in interpretation. These biases may compromise the reliability and validity of the findings, as participants might overestimate or underestimate their behaviors and experiences. Although our focus was on self-reported gambling behavior, we did not have access to fully validated scales measuring problem gambling behavior – in this we are limited by the original design of the study and data collection, which had a wide scope focusing on student health more generally (Raitasalo et al., 2024). Our results may also not generalize across different cultures and student populations. Given these limitations, our results should be viewed as somewhat tentative, but they are nonetheless an important step toward a comprehensive understanding of the factors driving gambling behavior.

In conclusion, our study offers three novel contributions to existing literature: (i) the role of study sector in gambling behavior (students in polytechnics, compared with university students, are more prone to gamble and experience harms); (ii) Self-perceived problem gambling was not significantly associated with drug use or smoking, but highly prominently with alcohol use; (iii) The somewhat surprising finding that compulsive internet use predicts problem gambling but not increased gambling activity. Future work should look more closely at the conceptual similarities and differences, particularly in student samples, between problem gambling and internet use, and their intercorrelations with drug use and smoking. Our results also highlight the need for increased awareness among those working with students for early detection of harmful behaviors. Gambling harm prevention efforts typically do not focus on students, which is not optimal given that students have a heightened risk for developing gambling disorder. Thus, screening for gambling disorder, or asking about potential issues with the students’ financial situations alongside excessive use of alcohol in student health checks may provide a valuable opportunity for early interventions (Huggett et al., 2021; Swanton and Gainsbury, 2020; Zolkwer et al., 2022).

## Data Availability

The datasets presented in this study can be found in online repositories. The names of the repository/repositories and accession number(s) can be found at the Finnish social science data archive (upon registration and request; https://services.fsd.tuni.fi/catalogue/FSD3616?lang=en&study_language=en).

## Footnotes

^1^The Finnish higher education system comprises universities and polytechnics (i.e., universities of applied sciences). Universities engage in education as well as research and can award doctorates. Polytechnics are multi-field institutions of professional higher education, and emphasize close contacts with business, industry and services, and the education has a pronounced occupational emphasis.
